# Interlocking Transcriptional Feedback Loops Control White-Opaque Switching in Candida albicans

**DOI:** 10.1371/journal.pbio.0050256

**Published:** 2007-09-18

**Authors:** Rebecca E Zordan, Mathew G Miller, David J Galgoczy, Brian B Tuch, Alexander D Johnson

**Affiliations:** 1 Department of Biochemistry and Biophysics, University of California San Francisco, San Francisco, California, United States of America; 2 Department of Microbiology and Immunology, University of California San Francisco, San Francisco, California, United States of America; Duke University Medical Center, United States of America

## Abstract

The human pathogen Candida albicans can assume either of two distinct cell types, designated “white” and “opaque.” Each cell type is maintained for many generations; switching between them is rare and stochastic, and occurs without any known changes in the nucleotide sequence of the genome. The two cell types differ dramatically in cell shape, colony appearance, mating competence, and virulence properties. In this work, we investigate the transcriptional circuitry that specifies the two cell types and controls the switching between them. First, we identify two new transcriptional regulators of white-opaque switching, Czf1 and white-opaque regulator 2 (Wor2). Analysis of a large set of double mutants and ectopic expression strains revealed genetic relationships between *CZF1, WOR2,* and two previously identified regulators of white-opaque switching, *WOR1* and *EFG1*. Using chromatin immunoprecipitation, we show that Wor1 binds the intergenic regions upstream of the genes encoding three additional transcriptional regulators of white-opaque switching *(CZF1, EFG1,* and *WOR2),* and also occupies the promoters of numerous white- and opaque-enriched genes. Based on these interactions, we have placed these four genes in a circuit controlling white-opaque switching whose topology is a network of positive feedback loops, with the master regulator gene *WOR1* occupying a central position. Our observations indicate that a key role of the interlocking feedback loop network is to stably maintain each epigenetic state through many cell divisions.

## Author Summary

The opportunistic fungal pathogen Candida albicans can switch between two heritable states—the “white” and “opaque” states. These two cell types differ in many characteristics, including cell structure, mating competence, and virulence. Recent studies of the molecular mechanism of regulating the white-opaque switch identified a master transcriptional regulator, Wor1. In this study, we identified two transcriptional regulators, Czf1 and Wor2, as new regulators of white-opaque switching. By constructing a series of single and double mutants and by examining where the master regulator Wor1 binds throughout the genome, we generated a molecular model of the bistable switch that regulates white-opaque switching. The regulatory model consists of interlocking positive feedback loops, which mutually reinforce one another and stabilize the opaque state. These results show how an organism can exist in two distinctive, heritable states without changes in the nucleotide sequence of its genome.

## Abstract 3

Analysis of mutations in*Candida albicans* indicates that the four genes in a circuit controlling white-opaque switching form a network of positive feedback loops, with the master regulator*WOR1* occupying a central position.

## Introduction

Transcriptional circuits are central to the regulation of many biological processes. Often the logic of the circuit, rather than the nature of its components, makes up its most critical feature. In this paper we describe an interlocking network of positive feedback loops that underlies white-opaque switching in the human fungal pathogen *Candida albicans*. White-opaque switching is an epigenetic phenomenon, where genetically identical cells can exist in two distinctive cell types, white and opaque [1]. Each cell type is stably inherited for many generations, and switching between the two types of cells occurs stochastically and rarely—roughly one switch in 10^4^ cell divisions [2]. The white form is the default cell type, and we propose that the main purpose of the network of interlocking feedback loops is, once excited, to stably maintain the opaque cell type through many cell divisions. Thus, we propose that the feedback loop network driving opaque formation is activated infrequently, but once activated, it is stably propagated through many cell generations.

Despite possessing the same genome, white and opaque cells have many phenotypic differences. Approximately 400 genes are differentially expressed between the two cell types, and the cells differ in their appearance under the microscope and in the color and shape of the colonies they produce on solid media [1,3,4]. They also differ in their behavior toward other C. albicans cells: opaque cells, but not white cells, are highly competent for mating; they respond to mating pheromone with polarized growth, and they can subsequently undergo cell and nuclear fusion with opaque cells of the opposite mating type [5–8]. Finally, the two types of cells appear to interact differently with their mammalian host, with opaque cells appearing more suited for skin infections, and white cells appearing to fare better in blood stream infections [9,10].

Several transcriptional regulators have been identified that play key roles in maintaining the white and opaque cell types, and in controlling the switching between them. Cells of mating type **a**/α are blocked for white-opaque switching, with all cells remaining locked in the white phase [5]. This block occurs through the action of two homeodomain proteins (a1 and α2), encoded at the *mating type-like a* (*MTLa)* and *MTLα* loci, respectively. The a1 and α2 proteins likely act together as a heterodimer to repress transcription of *WOR1,* the product of which is a positive regulator of the opaque state [11–13]. Wor1 is required for establishment and maintenance of the opaque state, and ectopically expressed *WOR1* drives cells into the opaque form. In **a** and α cells (both of which are permissive for switching), *WOR1* is expressed at low levels in white cells, but in opaque cells Wor1 activates its own synthesis, and *WOR1* expression levels rise dramatically. High levels of Wor1, produced by this positive feedback loop, are necessary to maintain the opaque state. Finally Efg1, which has been studied extensively for its role as regulator of the filamentous growth and pathogenesis in **a**/α (i.e., non-switching) strains of *C. albicans,* also plays a part in white-opaque switching: in **a** and α cells (but not **a**/α cells), cells deleted for *EFG1* exist almost exclusively in the opaque state [14,15] (this work). Thus *EFG1* is needed to stably maintain the white state.

In this paper we identify two additional transcription regulators of white-opaque switching, *CZF1* and *white-opaque regulator 2 (WOR2)*. The former has been previously studied as an important regulator of filamentous growth in **a**/α (non-switching) cells [16], but a role in white-opaque switching has not been previously described. *WOR2* has not been previously described in any context, and we named the gene *WOR2* based on its key role white-opaque switching, as described in this paper.

In order to understand the genetic relationships among *WOR1, EFG1, CZF1,* and *WOR2,* we constructed a large set of single and double mutants and analyzed them for white-opaque switching. We also ectopically expressed these regulators in mutant strains in various combinations and monitored their effects on switching and maintenance of the white and opaque states. Finally, we carried out chromatin immunoprecipitation (ChIP) experiments to establish direct regulatory connections between the central regulator, Wor1, and the other targets. We found that Wor1 binds upstream of: (1) all four transcriptional regulators investigated in this paper *(WOR1, CZF1, WOR2, EFG1);* (2) genes whose transcription is regulated by the white-opaque switch; and (3) a large number of genes that are not differentially transcribed during the white-opaque switch, suggesting an additional, previously unrecognized component of white-opaque switching, one that may require additional environmental inputs to fully reveal. Based on the combined results of these experiments, we have placed *MTLa1, MTLα2, WOR1, CZF1, WOR2,* and *EFG1* into a single genetic circuit regulating the white-opaque switch. This circuit is formed from a network of interlocking positive feedback loops, and we believe that this network can account for the stability of the white and opaque states through many cell generations.

## Results

### Identification of New Regulators of White-Opaque Switching

One of the most striking characteristics of the white-opaque switch is the large number of genes that are differentially regulated between the two types of cells. Approximately 400 genes have altered transcription; roughly half are up-regulated in the white phase, with the remaining half up-regulated in the opaque phase [3,4]. Several of these regulated genes encode transcriptional regulators, as predicted by the presence of a sequence-specific DNA-binding motif encoded in the gene. We tested a set of opaque-enriched transcription factors for possible roles in regulating the white-opaque switch.

Opaque-enriched genes were previously identified through microarray analyses that compared the gene expression profiles of isogenic white and opaque cells [3,4]. Genes up-regulated in opaque cells were searched for homology to transcriptional regulatory proteins using BLAST searches (http://www.ncbi.nlm.nih.gov/BLAST/) and Pfam motif searches (http://www.sanger.ac.uk/Software/Pfam/). From a set of 237 opaque-enriched genes, we chose to study six genes encoding putative transcriptional regulators: *CZF1, WOR2, HAP3,* orf19.4972, *CSR1,* and *PHO23*. Both *CZF1* and *WOR2* are predicted to contain a Zn(2)-Cys(6) zinc cluster motif, a known DNA-binding domain in fungal transcriptional regulators [17]; indeed *WOR2* had been provisionally named *ZCF33* to indicate it was the 33rd protein annotated with this zinc cluster motif. *HAP3* is predicted to contain a motif similar to the CCAAT-binding factor. The genes *CSR1* and orf19.4972 are predicted to each contain multiple C_2_H_2_ zinc fingers, a well-characterized DNA-binding domain. Because chromatin structure has been proposed to play a role in regulating the white-opaque switch [18], we also chose to investigate the opaque-enriched transcript *PHO23,* which encodes a protein containing a PHD domain and is predicted to be a part of the *RPD3* histone deacetylase complex.

For each of these candidate genes, we attempted to make homozygous deletion mutants in a white strain that is mating type **a**, and is thus permissive for switching to the opaque cell type (C. albicans is diploid, and it is therefore necessary to knock out two copies of each gene). Multiple independent deletion mutants of each target gene were made from independent heterozygous mutants. Despite numerous attempts, we were unable to create a homozygous knockout mutant of the *CSR1* gene and did not study *CSR1* further. White-opaque switching can occur in strains that are mating type **a** or α but not **a**/α. Most of the work presented in this paper was performed in **a** strains, but we know that Wor1 is also required for opaque formation in α strains (unpublished data and [13]), suggesting that white-opaque switching is controlled the same way in **a** and α strains. Consistent with this idea, a large set of microarray data indicates that the set of genes regulated by white-opaque switching is virtually identical in **a** and α cells [3].

For the five remaining candidates, we performed quantitative white-to-opaque switching assays as described previously [5] on at least two independent deletion mutants for each candidate gene, and multiple experiments were performed for each mutant (Table 1). As shown in Table 1, two mutants, the *czf1*Δ/*czf1*Δ and *wor2*Δ*/wor2*Δ knockouts, had a dramatic effect on white-opaque switching, forming opaque colonies much less frequently than did otherwise isogenic wild type (WT) **a** strains. The *CZF1* deletion strain formed opaque sectors and colonies ∼50-fold less frequently than WT **a** strains. The *wor2*Δ*/wor2*Δ mutant was never observed to form opaque colonies, representing a switching frequency at least 180-fold below that of the parent strain. Due to the key role this gene has in white-opaque switching, as described in this paper, we named the gene *WOR2*. These results implicate both *CZF1* and *WOR2* in the white-opaque switch; formally, they function as activators of the opaque state.

**Table 1:**
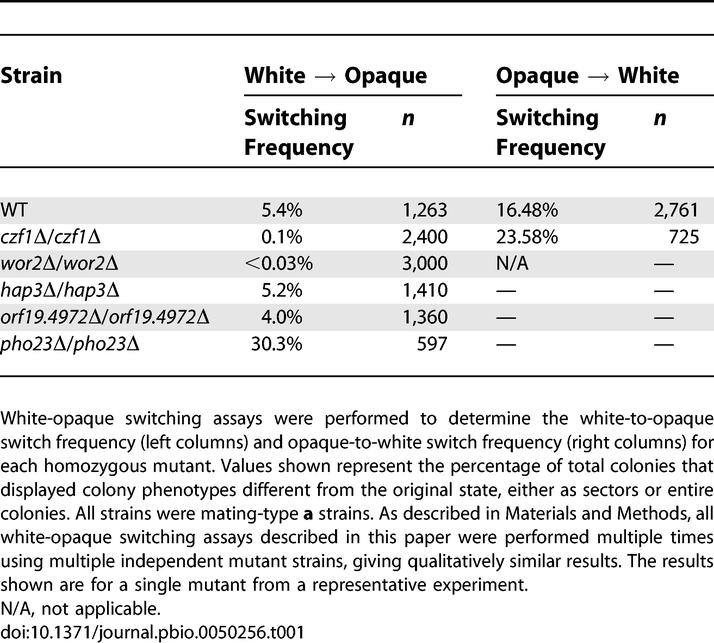
White-Opaque Switching Frequencies in Mutants Lacking Putative Opaque-Enriched Transcriptional Regulators

To verify that the defects in white-opaque switching were attributable to the disrupted genes, we complemented the *czf1*Δ/*czf*1Δ and *wor2*Δ/*wor2*Δ deletion mutants. Ectopic expression constructs, controlled by the *MET3* promoter were introduced into the *RP10* locus, as described previously [19]. Both the *czf1*Δ/*czf1*Δ *pMET3-CZF1* and *wor2*Δ/*wor2*Δ *pMET3-WOR2* strains were able to form opaque colonies when the *MET3* promoter was induced. However, when an empty vector was introduced, or the strains were grown on media that repressed the *MET3* promoter (and thus the only copy of *CZF1* or *WOR2*, respectively), the strains remained white. These results confirmed that the loss of *CZF1* or *WOR2* drastically reduces the ability for the strains to grow as opaque cells.

The deletion mutants lacking either orf19.4972 or *HAP3* formed opaque colonies at frequencies comparable to WT **a** strains and were not studied further. The *pho23*Δ/*pho23*Δ mutant switched to the opaque phase approximately six times as frequently as the WT control. If Pho23 works with Rpd3 in *C. albicans,* as is predicted based on homology in *Saccharomyces cerevisiae,* this result is consistent with a previous finding that *rpd3*Δ/*rpd3*Δ mutants have an increased frequency of interconversion between the white and opaque phases [18]. We did not study Pho23 further, because of its relatively small affect on switching frequencies, and because these effects could well be indirect: in *S. cerevisiae,* deletion of *RPD3* affects transcription levels of approximately 13% of the genome [20].

### Ectopic Expression of *CZF1,* but Not *WOR2,* Results in Opaque Formation in **a** Cells

We next expressed *CZF1* and *WOR2* ectopically in white cells to test whether either could drive white cells to the opaque form. All ectopic expression constructs described in this study were controlled by the *MET3* promoter integrated at the *RP10* locus, as previously described [19]. To test if ectopic expression of a given gene causes white-opaque switching, white strains were streaked from frozen stock onto repressing media and grown at room temperature for 1 wk. The strains were then plated for single colonies onto inducing media or repressing media, as a control, and grown for 1 wk at room temperature. The control **a** strain, with an empty vector (pCaEXP) inserted into the *RP10* locus, switched to the opaque phase at the typical low frequency, producing opaque sectors in approximately 0.5% of the colonies on both media conditions (Table 2), indicating that the media conditions used to control the *MET3* promoter do not significantly influence the frequency of white-opaque switching.

**Table 2:**
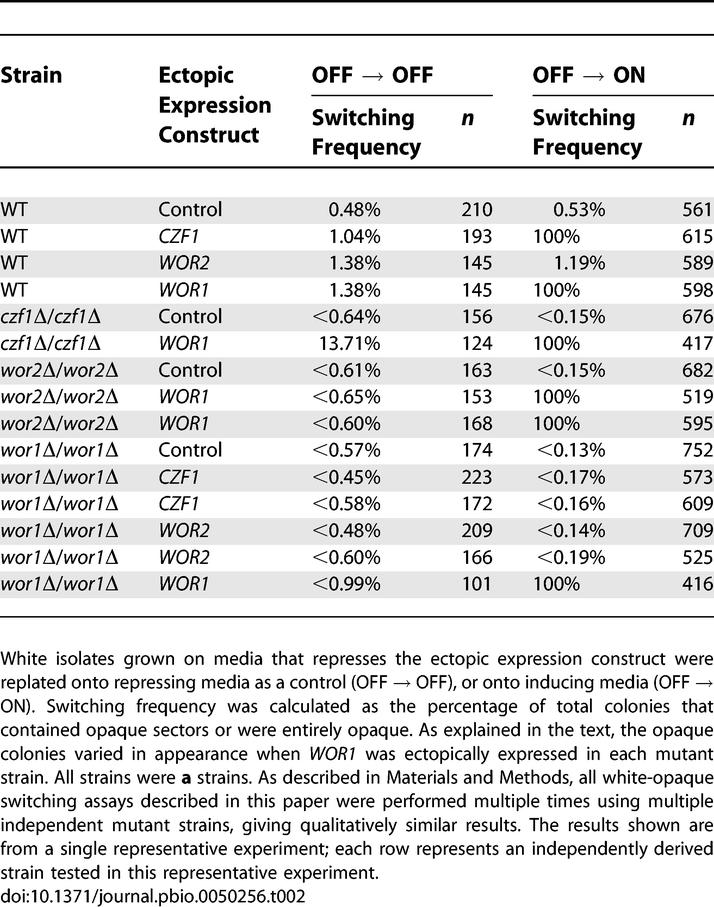
White-to-Opaque Switching Frequencies in Strains Ectopically Expressing *CZF1, WOR1,* or *WOR2*

We found that ectopic expression of *CZF1* in WT **a** cells led to a mass conversion to the opaque phase (Table 2), but only when the *MET3* promoter was induced. In contrast, expression of a *pMET3-WOR2* construct did not drive the white-to-opaque switching; the cells remained white, based on colony appearance (Table 2) and cell shape (unpublished data). We know that the *pMET3-WOR2* construct is functional from the complementation studies described earlier, thus this result indicates that the ectopic expression of *WOR2,* at least at the level driven by the *MET3* promoter, is not sufficient to drive opaque formation in an otherwise WT **a** strain.

### Genetic Epistasis Shows that *WOR1* Is Downstream of *CZF1* and *WOR2* in the Regulation of the White-Opaque Switch

Previous work on the regulation of white-opaque switching identified *WOR1* as a master regulator of the white-opaque switch [11–13]. In the next set of experiments, we tested the genetic interactions between *WOR1, CZF1,* and *WOR2* in order to understand how they work together to regulate the switch. As is the case for *CZF1* and *WOR2,* deletion of *WOR1* drastically reduces the frequency of opaque formation. Like *CZF1,* ectopic expression of *WOR1* causes mass conversion to the opaque phase in otherwise WT **a** cells.

**Figure 1:**
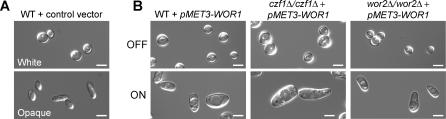
Ectopic Expression of *WOR1* Drives Opaque Formation in *czf1*Δ/*czf1*Δ or *wor2*Δ/*wor2*Δ Strains Images are differential interference contrast photomicrographs of cells resuspended from colonies. (A) White and opaque isolates of WT strains with an empty control vector, grown on media that induces the *MET3* promoter (ON). (B) Ectopic expression of *WOR1* in WT, *czf1*Δ/*czf1*Δ, or *wor2*Δ/*wor2*Δ strains; cells were grown on media to repress (OFF) or induce (ON) the *pMET3-WOR1* construct. All strains are **a** strains. Scale bar = 5 μm.

We first expressed *WOR1* ectopically in a *czf1*Δ/*czf1*Δ or *wor2*Δ*/wor2*Δ **a**-cell strain and observed the effects on white-opaque switching. We found that when *WOR1* was ectopically expressed in white *czf1*Δ/*czf1*Δ mutants, most of the colonies grew in the opaque phase or had opaque sectors (Table 2), although the colonies had a slightly rougher texture than did conventional opaque colonies on inducing media (unpublished data). Inspection of the cells from the opaque colonies revealed elongated cells, typical of opaque cells (Figure 1). In a control experiment, a *czf1*Δ/*czf1*Δ mutant with pCaEXP, an expression vector lacking *WOR1,* was not converted into opaque cells (Table 2).

When we expressed *WOR1* ectopically in a white *wor2*Δ*/wor2*Δ **a** strain, we observed that all of the colonies contained cells that had switched to the opaque phase (Figure 1), usually in the form of opaque sectors, though the opaque sectors were slightly lighter in color than those of normal opaques (Table 2). This strain was never observed to form opaque cells when grown on media that repressed expression of the ectopic *WOR1*. The *wor2*Δ*/wor2*Δ strain with pCaEXP, an empty expression vector, also appeared locked in the white phase, whether it was grown on the repressing or inducing media (Table 2).

Next, we tested the effects of ectopic expression of *CZF1* in a *wor1*Δ*/wor1*Δ **a** strain. We found that these strains remained locked in the white phase (Table 2); they were indistinguishable from a *wor1*Δ*/wor1*Δ mutant. Finally, we tested the ectopic expression of *WOR2* in a *wor1*Δ*/wor1*Δ **a** strain, and we found no change in the switching frequency, as compared to a *wor1*Δ*/wor1*Δ mutant (Table 2). This result was expected, given that induction of the *WOR2* ectopic construct had no effect in a WT background.

Taken together, these results indicate that *CZF1* and *WOR2* function upstream of *WOR1;* thus ectopic expression of *WOR1* suffices for opaque cell formation whether or not *WOR2* and *CZF1* are present. However, the converse is not true: deletion of *WOR1* cannot be overcome by ectopic expression of *CZF1*.

### *CZF1* Contributes to Formation of the Opaque State, but Is Not Necessary for Heritability of the Opaque State

Unlike *wor1*Δ/*wor1*Δ and *wor2*Δ/*wor2*Δ mutants, *czf1*Δ/*czf1*Δ mutants do form opaque colonies, albeit infrequently. As described above, ectopic *CZF1* expression can induce a switch to the opaque state. To clarify *CZF1*'s role in white-opaque switching, we examined switching in the reverse direction, where opaque cells switch to white cells. When opaque isolates of WT **a** strains were replated on repressing media, about 16% of the colonies switched back to the white form (Table 1). The rare opaque isolates of *czf1*Δ/*czf1*Δ **a** strains were nearly as stable as WT opaques; upon replating, 23% of the colonies contained white cells.

Because opaque isolates in *czf1*Δ/*czf1*Δ strains are rare, we sought to create more opaque *czf1*Δ/*czf1*Δ isolates in order to test the stability of the opaque cells lacking Czf1. To do this, we used the *pMET3-WOR1* ectopic expression construct to drive *czf1*Δ/*czf1*Δ strains to the opaque state, as described above. When the *pMET3-WOR1* construct was subsequently repressed in the *czf1*Δ/*czf1*Δ opaque **a** strains, at least 92% of the colonies remained opaque (Table 3). Similarly, a pulse of *pMET3*-*WOR1* in WT white **a** cells is sufficient to generate stable opaque populations; the ectopic Wor1 expression can be repressed and the strains will largely continue to grow in the opaque phase (Table 3) [11]. These data indicate that, although its presence is important to form opaque cells, Czf1 contributes minimally to the stability (that is, the heritability) of the opaque state, once it has been established.

**Table 3:**
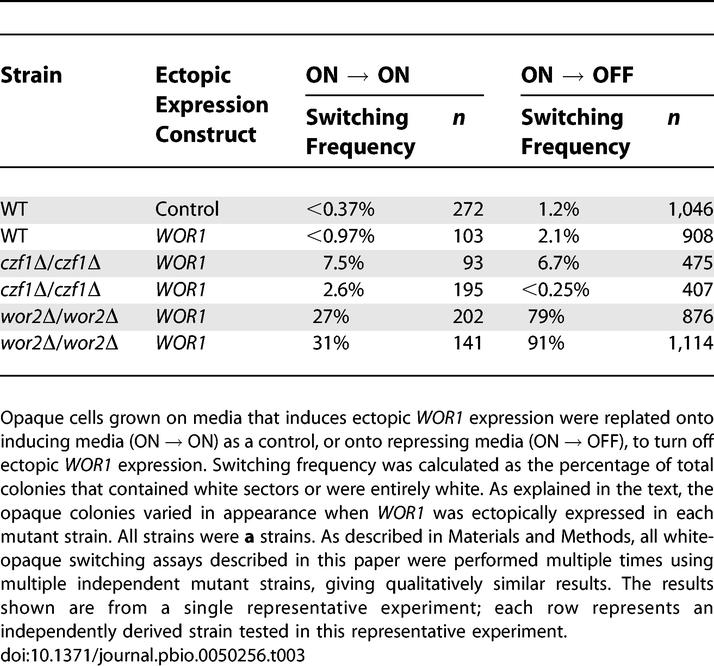
Stability of the Opaque State when Ectopic Expression of *WOR1* Is Repressed

### *WOR2* Is Necessary for the Stability of the Opaque State

In parallel with the studies described above, we examined *WOR2*'s role in maintaining the heritability of the opaque state. As described, when *WOR1* was ectopically expressed in *wor2*Δ*/wor2*Δ mutants, opaque cells formed. When the *pMET3-WOR1* construct was then repressed in these cells (Table 3), the majority of the cells reverted to the white form (Table 3). In contrast, *WOR2/WOR2* control strains remained in the opaque form for many generations after the pulse of *WOR1* expression. These results indicate that Wor2 contributes greatly to the stability of the opaque state, once it has been formed.

### Deletion of *EFG1* Causes Opaque Formation in **a**, but Not **a**/α Strains

Thus far, we have only considered the role of the opaque-enriched transcription factors *WOR1, CZF1,* and *WOR2* in the regulation of the white-opaque switch. However, a fourth regulator, *EFG1,* which is up-regulated in white cells, is known to participate in white-opaque switching [14,15]. Experiments reported by Sonneborn et al. [14] suggested that depletion of Efg1 induced the formation of opaque cells in some **a**/α strain backgrounds, but not in others. To clarify these results, we constructed new isogenic homozygous *efg1*Δ/*efg1*Δ mutants in **a** or **a**/α strains. In the mating type **a** strain, we found the *efg1*Δ/*efg1*Δ mutation caused a majority of the population to switch to the opaque phase; over 98% of the colonies contained opaque sectors (Table 4), with many colonies showing multiple sectors. We also observed a small number of entirely white colonies, indicating that *EFG1* is not strictly necessary for growth in the white phase. We also examined the opaque-to-white switching frequency in *efg1*Δ/*efg1*Δ mutants; we found that they switched to the white phase ∼80 times less frequently than WT **a** strains (Table 4). Thus, deletion of *EFG1* dramatically increased the likelihood the cells will grow in the opaque phase, confirming previous studies in WO-1, an α strain [15].

**Table 4:**
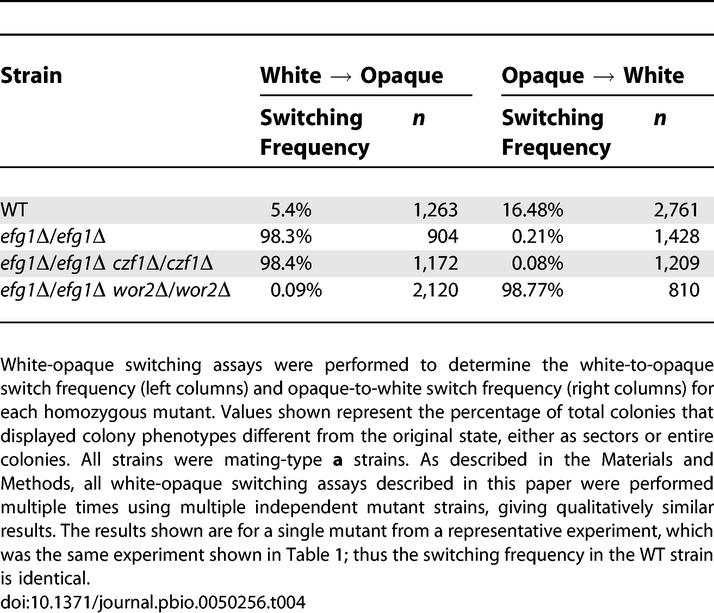
Epistasis among *EFG1, CZF1,* and *WOR2* in the Regulation of White-Opaque Switching

**Figure 2:**
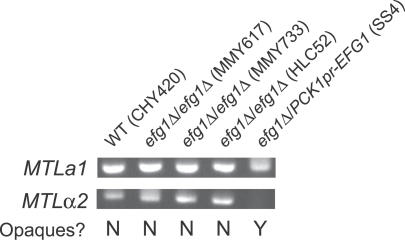
Verification of Mating Type in *efg1*Δ/*efg1*Δ Strains Whole-cell PCR was performed to verify the presence of the *MTLa1* and *MTLα2* genes in a series of *efg1*Δ/*efg1*Δ mutants. As described in Materials and Methods, PCR amplification of the **a** and α alleles of each of the five genes present at the mating type locus confirm these results. The ability of each mutant to form opaque colonies, as described in the Results, is indicated with Y (yes) or N (no).

In contrast to the previous reports, we never observed opaque colonies or sectors in the *efg1*Δ/*efg1*Δ mutant in an **a**/α strain, despite observing over 3,200 colonies (unpublished data). We obtained the previously published *efg1*Δ/*PCK_pr_*-*EFG1* **a**/α mutant that showed opaque cell formation when remaining allele of *EFG1* was repressed [14]. Using PCR to amplify the *MTLa1* and *MTLα2* genes, we determined that this mutant was an **a** strain, likely due to spontaneous loss of one copy of Chromosome 5, which carries the *MTL* locus (Figure 2). Additionally, each of the *efg1*Δ/*efg1*Δ mutants that were locked in the white phase was confirmed to be an **a**/α strains (Figure 2). These results indicate that the loss of Efg1 from **a** cells causes massive conversion to the opaque state, but this conversion is blocked in **a**/α cells. Thus Efg1 functions upstream of the a1-α2 block of white-opaque switching.

### Epistasis among *EFG1, CZF1,* and *WOR2*

To understand the genetic interplay between *EFG1, CZF1,* and *WOR2*, we created strains that lacked the white-enriched regulator *EFG1* and each of the opaque-enriched regulators, *CZF1* or *WOR2*. These double homozygous knockouts were then tested in quantitative switching assays and monitored for the frequency of forming white, opaque, and sectored colonies. Nearly all colonies of the *efg1*Δ/*efg1*Δ *czf1*Δ/*czf1*Δ mutant were in the opaque phase or contained opaque sectors (Table 4), reflecting the phenotype of the *efg1*Δ/*efg1*Δ mutant. When the opaque colonies isolated from *efg1*Δ/*efg1*Δ *czf1*Δ/*czf1*Δ were replated to test the heritability of the state, only 0.08% of the colonies returned to the white state, in comparison with the normal opaque-to-white switching frequency, where ∼16% of the colonies are white or have white sectors. Thus, the *efg1*Δ/*efg1*Δ *czf1*Δ/*czf1*Δ opaque cells are approximately 200-fold more stable than WT opaque cells, a stability similar to that of *efg1*Δ/*efg1*Δ **a** mutants (Table 4). Thus, in both forward and reverse switching frequency, the *efg1*Δ/*efg1*Δ *czf1*Δ/*czf1*Δ double mutants closely resembled the *efg1*Δ/*efg1*Δ single mutant.

We also examined the switching behavior of an *efg1*Δ/*efg1*Δ *wor2*Δ*/wor2*Δ mutant. In this strain, white colonies accounted for ∼99% of the total colonies seen, reflecting the phenotype of the *wor2*Δ*/wor2*Δ mutant. We also tested the stability of these rare opaque colonies that were formed by the *efg1*Δ/*efg1*Δ *wor2*Δ*/wor2*Δ mutant. When replated, these opaque cells proved to be highly unstable; over 98% of the colonies were white or contained white sectors. Thus, in both forward and reverse switching, the *efg1*Δ/*efg1*Δ *wor2*Δ*/wor2*Δ double mutant resembled the *wor2*Δ*/wor2*Δ mutant.

### Wor1 Is Bound to DNA Upstream of *CZF1, WOR2,* and *EFG1*

The genetic epistasis data presented above places *WOR1* at the center of white-opaque regulation; formally, it is the most “downstream” regulator of opaque formation, as its deletion blocks white-opaque switching in all contexts tested. Moreover, ectopic *WOR1* expression suffices to switch white cells to opaque cells when any of the other opaque-enriched transcription factors are deleted. Previous work indicated that Wor1 expression is maintained through an auto-stimulatory positive feedback loop, mediated by Wor1 binding at its own promoter [11]. Expression of the genes encoding Czf1, Wor2, and Efg1 are all regulated by white-opaque switching; in the opaque form, *CZF1* and *WOR2* are up-regulated, and *EFG1* is down-regulated relative to the white form. Thus, in a formal sense all three are regulated by Wor1.

To test whether Wor1 directly regulates *CZF1, WOR2,* and *EFG1*, we performed ChIP using an affinity purified antibody (α-Wor1_Nterm_), raised against a peptide near the N terminus of Wor1. These ChIPs were analyzed genome-wide using microarrays (ChIP-chip): the precipitated DNA was amplified, fluorescently labeled, and competitively hybridized against genomic DNA (input DNA) on custom DNA tiling microarrays containing 60-mer oligonucleotides tiled at 80 bp intervals across the entire C. albicans genome. Two microarrays were hybridized with DNA from two separate immunoprecipitations (IPs) of an opaque WT **a** strain; a single ChIP was performed in a *wor1*Δ*/wor1*Δ (white) **a** strain as a control.

We examined the Wor1 ChIP data using previously published software that implements a statistical model and integrates data from several neighboring spots along chromosomes to identify IP enrichment peaks [21]. Using standard parameters, this procedure identified 206 peaks of Wor1 enrichment across the genome in opaque cells. These peaks of Wor1 enrichment were confirmed by visual inspection of ChIP-chip data plotted along chromosomes. Of these peaks, 25 also appeared in the ChIP-chip of a *wor1*Δ/*wor1*Δ strain, and likely represent cross reactivity or particularly “sticky” proteins; they were removed from further analysis, leaving a set of 181 peaks of Wor1 enrichment. In parallel, we performed a series of ChIP-chip experiments using a different antibody against Wor1 (raised against a peptide at the C terminus of the Wor1 protein, α-Wor1_Cterm_) and identified 122 peaks of Wor1 enrichment in opaque strains that were not detected in the *wor1*Δ/*wor1*Δ control strain. By comparing the sets of peaks of Wor1 enrichment identified using both antibodies, we found that 112 peaks are enriched for Wor1 in opaque cells (but not *wor1*Δ/*wor1*Δ strains) using both antibodies. Thus, 112/122 (92%) of the peaks identified using the α-Wor1_Cterm_ were also found using the N-terminal antibody, indicating the set of peaks identified with α-Wor1_Cterm_ is almost entirely a subset of α-Wor1_Nterm_ ChIP-chip data. We found that 112/181 (62%) of the targets identified in using the α-Wor1_Nterm_ antibody were also detected using the α-Wor1_Cterm_ in opaque cells. Because the experiments using α-Wor1_Nterm_ exhibited very little cross-reactivity in *wor1*Δ/*wor1*Δ strains and virtually encompassed the set found using α-Wor1_Cterm_, we chose the targets identified in the α-Wor1_Nterm_ ChIP-chip as our set of high-confidence Wor1 targets for further analysis.

With 181 peaks identified as high confidence Wor1 targets using the α-Wor1_Nterm_ antibody, we turned to the question of identifying the genes potentially regulated by Wor1. We chose to limit the set to the 170 peaks positioned in intergenic regions upstream of at least one open reading frame (ORF); this eliminated three peaks positioned within ORFs and eight peaks positioned between convergent ORFs. Because some of the 170 peaks of Wor1 enrichment lay in the intergenic region of divergently transcribed genes, there are 221 genes potentially regulated by Wor1 (Table S1).

**Figure 3:**
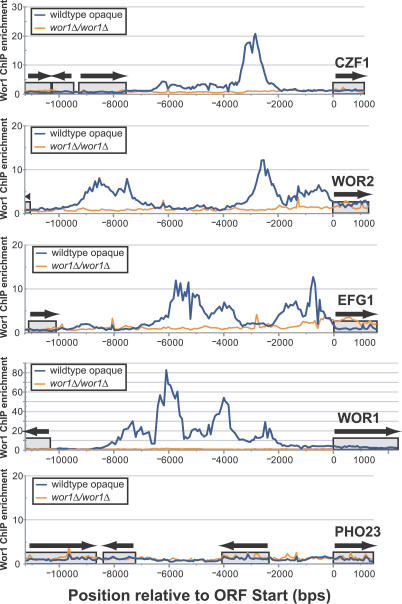
Wor1 Binds Upstream of the *CZF1, WOR2, EFG1,* and *WOR1* Genes ChIP was performed with α-Wor1_Nterm_ antibodies in a WT opaque **a** strain or a *wor1*Δ/*wor1*Δ (white) **a** strain. The ChIP enrichment was detected by hybridization to a C. albicans tiling microarray with probes every ∼80 bp across the genome. The top three binding profiles show Wor1 enrichment upstream of the *CZF1, WOR2, and EFG1* ORFs. The fourth binding profile serves as a positive control of Wor1 enrichment, seen at the *WOR1* promoter [11]. The bottom binding profile, showing the DNA upstream of *PHO23*, serves as an example of the low levels of Wor1 enrichment seen throughout the majority of the genome. Grey boxes indicate ORFs and arrows indicate the orientation of each coding sequence along the chromosome.

We found clear Wor1 enrichment at the intergenic regions immediately upstream of the *CZF1, WOR2,* and *EFG1* coding sequences (Figure 3). In the ∼7.6 kb of intergenic sequence upstream of *CZF1,* we found segments that were enriched up to 20-fold for Wor1, as compared to a *wor1*Δ*/wor1*Δ control strain. Upstream of the *WOR2* coding sequence, we found segments enriched up to 11-fold. Wor1 was enriched up to ∼12-fold in the 10.1 kb upstream of *EFG1*. We also verified that Wor1 was found upstream of the *WOR1* gene using the tiling arrays, showing enrichment up to ∼80-fold in the opaque cells, as compared to the control *wor1*Δ/*wor1*Δ strain (Figure 3) [11]. These results confirm that Wor1 is present at its own promoter in opaque cells, and reveal that Wor1 is also present at the promoters of *CZF1, WOR2,* and *EFG1* in opaque **a** cells.

We also compared the set of 221 genes potentially regulated directly by Wor1 to the set of genes differentially transcribed between white and opaque cells [3]. We found Wor1 enrichment at the intergenic regions upstream of 38 opaque-enriched genes and 20 white-enriched genes (Table S1), suggesting that Wor1 directly controls expression of approximately 15% of the genes regulated by white-opaque switching. These results also suggest that Wor1 may function in opaque cells as both a transcriptional repressor and as an activator. Though there is some ambiguity in ascribing Wor1 regulation at divergently transcribed genes, we estimate that Wor1 also binds more than 100 genes that have not been previously identified as white- or opaque-enriched by transcriptional profiling.

As described above, Wor1 protein is bound at the intergenic DNA upstream of the genes *WOR1, EFG1, CZF1,* and *WOR2*. Each of these genes has a remarkably long upstream region of DNA (at least 7 kb in each case), and Wor1 appears to be bound at multiple positions along these regions. From the ChIP-chip experiments, we found that occupancy of large intergenic regions is a general characteristic of Wor1. Analysis of the intergenic regions at all 6,077 gene promoters in the C. albicans genome (excluding those at telomeres) revealed a median promoter length of 623 bp, whereas the median promoter length of the 181 gene promoters bound by Wor1 was 3,390 bp (unpublished data). This preference is especially pronounced when considering the intergenic regions over 10 kb; Wor1 enrichment was seen at 12 of 19 of these intergenic regions. Intriguingly, over half (seven of 12) of these intergenic regions lie upstream of genes encoding sequence-specific DNA binding proteins—*WOR1, RFG1, TCC1, WOR2, ZCF37, EFG1,* and *RME1*—suggesting Wor1 may exert much of its control over the white-opaque switch indirectly through other transcriptional regulators.

## Discussion

**Figure 4:**
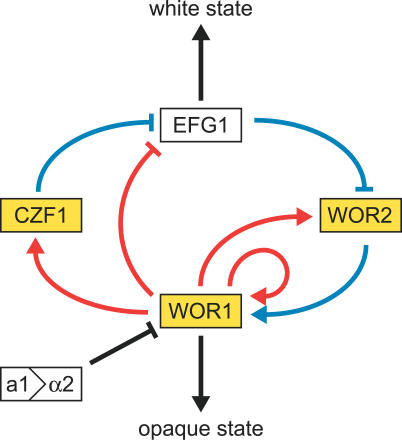
Model of the Genetic Network Regulating the White-Opaque Switch White and gold boxes represent genes enriched in the white and opaque states, respectively. Blue lines represent relationships based on genetic epistasis. Red lines represent Wor1 control of each gene, based on Wor1 enrichment in chromatin immunoprecipitation experiments. Activation (arrowhead) and repression (bar) are inferred based on white- and opaque-state expression of each gene.

**Figure 5:**
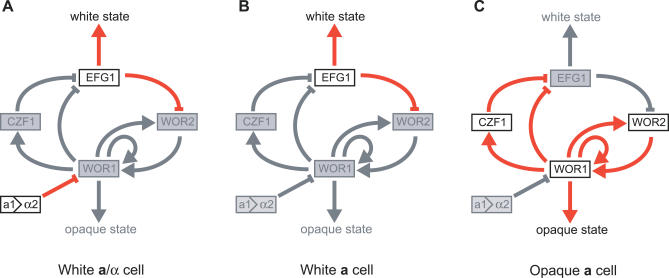
Activity of the White-Opaque Genetic Regulatory Network in Different Cell Types In each scenario, genes indicated by white boxes are up-regulated and genes in gray boxes are down-regulated. Red lines represent active regulatory relationships; gray lines represent relationships that are inactive, due to the down-regulation of the effector gene. (A) In white **a**/α cells, the a1-α2 heterodimer represses *WOR1,* keeping the Wor1-mediated feedback loops inactive. This allows *EFG1* expression and formation of white cells. (B) In white **a** cells, *EFG1* expression contributes to the formation of white cells and down-regulates *WOR2*. This helps keep Wor1 expression low in white cells, even though the a1-α2 repression of *WOR1* has been lifted. (C) In opaque **a** cells, *WOR1* expression levels are up-regulated, which in turn activates the represented positive feedback loops, the net effect being increased *CZF1, WOR2,* and *WOR1* expression, and decreased *EFG1* expression.

In this paper, we have dissected the genetic circuitry controlling white-opaque switching in the fungal pathogen *C. albicans*. White-opaque switching is an epigenetic change between two distinct types of cells, both containing the same genome. The white-opaque switch is crucial for many aspects of C. albicans biology, including interactions with other C. albicans cells (pheromone sensing and mating) and interactions with the host (opportunistic pathogenesis). Our results are summarized in Figures 4 and 5, where the circuitry controlling this switch is diagrammed. This network of positive feedback loops is responsible for the heritability of each state, as well as the frequency of switching between them, and we propose that the structure of this network makes an important contribution to the biology of white-opaque switching.

The default state can be considered the white cell type: most clinical isolates of *C. albicans* are **a**/α cells, and they are locked in the white state through a1-α2 repression of *WOR1* (Figure 5A). However, even in **a** and α cells, which are permissive for white-opaque switching, the white cell type still seems to be the default, in that white cells are generally more stable than opaque cells (Figure 5B). For example, opaque cells at 24 °C are stable for many generations, but above 30 °C they become unstable and rapidly switch back en masse to the white form, which is stable under these conditions [1,2]. There are no known environmental conditions that comparably destabilize the white form. In our model, the opaque form is generated when the series of positive feedback loops shown in Figure 4 become excited (Figure 5C). Thus, in opaque cells, *WOR1* likely directly induces *CZF1* and *WOR2* expression, and in turn, *CZF1* and *WOR2* both activate *WOR1*. *CZF1* does this by repressing a repressor of the opaque state (*EFG1*), the net effect being a positive feedback loop.

The multiple feedback loops observed in the opaque state are reminiscent of those seen in differentiated animal cells, such as those of the *Drosophila* eye ([22], reviewed in [23]) and the mammalian myoblast ([24], reviewed in [25]). A series of such feedback loops (as opposed to a single loop) buffers the circuit against transient fluctuations in any single regulatory protein and therefore provides additional stability to the excited form of the circuit. In addition, the nature of the circuit probably defines the switching frequency. For example, deletion of *CZF1* decreases the white-to-opaque switching frequency by approximately 10-fold, but has little effect on the backward switching rate. Thus, the primary role of *CZF1* seems to be in modulating the switching frequency; in contrast, *WOR1* and *WOR2* are both required to maintain the opaque state; thus their roles are more integral to the switch itself.

Although the overall logic of the circuit shown in Figure 4 can explain many features of white-opaque switching, there appear to be several unusual features of the circuit components themselves that likely also play important roles in white-opaque switching. For example, our ChIP-chip experiments revealed that Wor1 binding shows a bias toward genes with unusually long upstream intergenic regions—as defined by the distance from the 5′ end of the ORF to the next annotated coding region. This observation suggests that these genes bound by Wor1, which include the four encoding transcriptional regulators that form the interlocking feedback loops (*WOR1*, *EFG1*, *CZF1*, and *WOR2*) are also controlled by a number of other transcriptional regulators. It is known that the frequency of white-opaque switching can be influenced by environmental cues (e.g., temperature and oxidative stress) [1,26], and it seems plausible that different rates of switching could be “set” by individually adjusting the levels of the regulatory proteins that make up the circuit. For example, since deletion of *CZF1* reduces the frequency of white-to-opaque switching 10-fold, regulation of the level of Czf1 by environmental signals could directly control the “forward” switching rate. Another unusual feature of the circuit concerns the wide distribution of Wor1 over much of the upstream regions of *WOR1, EFG1, CZF1,* and *WOR2* (Figure 3), suggesting a highly cooperative transcriptional response to the intracellular levels of *WOR1*. This, combined with the interlocking positive feedback loops, could be responsible for the switch-like behavior of the system, specifically the failure to readily observe a cell type intermediate between white and opaque in wild-type switching strains.

The switch from the white to the opaque form growth alters transcription of approximately 400 genes. We know that the master regulator Wor1 ultimately controls all of these genes, since deletion of *WOR1* locks cells in the white form, and ectopic expression of *WOR1* converts white cells en masse to opaque cells. Our ChIP-chip analysis revealed that Wor1 directly regulates approximately 15% of this gene set (20 white-enriched genes and 38 opaque-enriched genes). Since Wor1 is also bound upstream of *CZF1, EFG1, WOR2,* and 20 additional transcriptional regulators (see Table S1), it seems likely that much of white-opaque switching program is regulated indirectly by Wor1 through its effects on other transcriptional regulators.

An unexpected outcome of the Wor1 ChIP-chip experiments was the presence of Wor1 at a large number of genes that were not identified as white- or opaque-enriched in previous microarray analyses [3]. There are several explanations for this observation. First, Wor1 could control these genes in both white and opaque cells, with their transcription being unaffected by the white-to-opaque transition. We think this explanation is unlikely because Wor1 is up-regulated 45-fold in opaque cells [3], and it seems improbable that this change could have no impact on expression of target genes. To test this idea directly, we performed a Wor1 ChIP-chip experiment in white **a** cells, and found that Wor1 is not bound at any of these target genes (unpublished data). A second possibility, one that we favor, is that Wor1 may occupy the promoters of these 100 genes in opaque cells, preparing their expression to respond to unknown environmental signals, perhaps those generated by the host. According to this idea, the standard laboratory conditions used for transcriptional profiling would not have included the necessary environmental stimuli, and thus these genes would not have been identified as regulated by the white-opaque switch. This idea suggests there are additional aspects to white-opaque switching which have not been previously recognized.

Finally we note that white-opaque switching does not appear to be a general feature of fungi, even those that are closely related to C. albicans. Indeed it may have arisen during the long association of C. albicans with its warm-blooded hosts. The evolution of a complex circuit composed of interlocking feedback loops is relatively simple to imagine, as it could occur stepwise simply through the acquisitions of cis-acting sequences in genes for transcriptional regulators used for other purposes in the cell. We note that Czf1 also relieves Efg1-mediated repression of hyphal growth under embedded conditions [27], and this genetic relationship has been maintained in the regulation of the white-opaque switch. Thus *EFG1* and *CZF1* have other key functions in the cell—even in cells that are genetically blocked for white-opaque switching—and their involvement in white-opaque switching could well be a recent adaptation, functioning to modulate the stability of the two states and the frequency of switching between them. The independent evolution of interlocking transcriptional feedback loops in a variety of distinct biological contexts (white-opaque switching in *C. albicans,* eye development in flies, and muscle development in mammals, for example) suggests they are particularly effective ways of providing, from the same genome, distinctive cell types that can be stably propagated for many generations.

## Materials and Methods

All strains and primers used in this study are listed in Tables S2 and S3, respectively. DNA sequences of C. albicans genes were obtained from the *Candida* Genome Database (http://www.candidagenome.org/)

### Media.

Standard laboratory media have been described previously [28]. Synthetic complete media, supplemented with 2% glucose and 100 μg/ml uridine (SCD+Urd) was used to maintain strains in the white and opaque phases at room temperature. For ectopic expression experiments, cells were grown on inducing media (SCD−Met−Cys+Urd) or repressing media (SCD+Met+Cys+Urd) to control expression of the *MET3* promoter, as described previously [11,19].

### Plasmids.

The plasmid containing the *pMET3-WOR1* construct (pRZ25) has been described before [11]. To make the *pMET3-WOR2* and *pMET3-CZF1* constructs, the *WOR2* or *CZF1* ORFs was PCR-amplified from SC5314 genomic DNA using primers containing BamHI and SphI restriction sites, and cloned into a BamHI/SphI-digested pCaEXP, to create the plasmids pAJ2230 and pAJ2231, respectively.

### Strain construction.

All strains were derived from SC5314. *EFG1, CZF1,* or *WOR2* was deleted using a modified Ura-blaster protocol [29]. In short, the recyclable *URA3-dpl200* marker was PCR-amplified from pDDB57 using long oligonucleotides identical to the sequence immediately flanking each ORF targeted for deletion. The deletion construct was transformed into CHY439 (*α1*Δ*α2*Δ, Ura^−^) or CAI4 (**a**/α, Ura^−^) and transformants were selected on SD−Ura media. 5-Fluoro-orotic acid was used to counterselect against *URA3* marker, and the resulting Ura^−^ isolates were used for subsequent rounds of gene deletion or to create the ectopic expression strains. For each knockout target, at least two homozygous deletion mutants were created from independent heterozygous mutants. When creating double mutants, *CZF1* and *WOR2* were each deleted in an *efg1*Δ/*efg1*Δ (*α1*Δ*α2*Δ, Ura^−^) mutant. In the case of the *efg1*Δ/*efg1*Δ *wor2*Δ/*wor2*Δ mutant, two independent double mutants were created from two independent *efg1*Δ*/efg1*Δ homozygous deletion mutants. Each *WOR1* allele was deleted from the strain SNY78 (**a**/α, His^−^, Leu^−^, Ura^−^) using fusion knockout constructs described previously [11]. The resulting strain was grown on sorbose-containing media to generate **a**/**a** strains (see [6] and references therein), creating the *wor1*Δ/*wor1*Δ (**a**/**a**, Ura^−^) strain.

Ectopic expression constructs pAJ2230, pAJ2231, pRZ25 (described above), or pCaEXP (empty control vector [19]) were linearized to direct integration to the *RP10* locus and transformed into Ura^−^ isolates of WT, *wor2*Δ/*wor2*Δ, *czf1*Δ/*czf1*Δ, or *wor1*Δ/*wor1*Δ strains. To create the duplicate ectopic expression strains listed in Table S2, ectopic expression constructs were introduced into independent *wor1*Δ/*wor1*Δ or *wor2*Δ/*wor2*Δ strains. The *czf1*Δ/*czf1*Δ (Ura^−^) strains used to create the *czf1*Δ/*czf1*Δ + *pMET3-WOR1* ectopic expression strains are different Ura^−^ loopout isolates generated by 5-fluro-orotic acid counter-selection of the same *czf1*Δ/*czf1*Δ (Ura^+^) strain. The *czf1*Δ/*czf1*Δ + *pMET3-CZF1* complementation strains were made from the same *czf1*Δ/*czf1*Δ knockout strain.

### Basic white-opaque switching assays.

Switching frequencies between the white and opaque phases were determined in plate-based assays, as described previously, with modifications [5]. Strains were streaked from frozen stock onto SCD+Urd and grown at RT for 5–7 d. For each strain, at least five entirely white colonies were resuspended into dH_2_O, diluted, and plated for single colonies on SCD+Urd. After growth at RT for 1 wk, we examined the colonies and counted the number of switch events (as evidenced by the presence of opaque sectors, or entirely opaque colonies). The same process was used to assess opaque-to-white switching, but the original frozen stocks contained opaque isolates of each strain, and we monitored switching by the presence of white sectors or entirely white colonies. The data shown in Tables 1 and 4 were taken from the same representative experiment and only tested one strain of each genotype. In repetitions of the switching assays (unpublished data), multiple independent deletion mutants of each genotype were tested and yielded results similar to those shown in Tables 1 and 4.

### White-opaque switching using ectopic expression constructs and cell images.

Switching assays in strains containing the *pMET3* ectopic expression constructs were performed as described in [11], with modifications. In short, to test if ectopic expression can drive opaque formation, white strains were streaked from frozen stock onto repressing media at RT for 5 d. At least five fully white colonies were replated for single colonies on inducing media (or repressing, as control). After growth at RT for 1 wk, colony phenotypes were recorded. Colonies were resuspended in sterile water and cells were examined by using differential interference contrast microscopy on an Axiovert 200M microscope (Carl Zeiss, http://www.zeiss.de/). All experimental strains, excepting the *wor1*Δ/*wor1*Δ + *pMET3-WOR2* strains, were tested in at least two repetitions of the switching assay. Data shown in Table 2 are from a single representative experiment, and each strain listed is an independent ectopic expression mutant.

To test if the resulting colony phenotypes were stable after the ectopic expression was repressed, opaque strains (formed by induction of the ectopic expression constructs) were streaked from frozen stock onto inducing media at room temperature. At least five opaque colonies were resuspended in sterile dH_2_O and replated onto repressing media (or inducing, as control) and grown at RT for 1 wk. Colony phenotypes were recorded. Two independently derived strains were tested for each ectopic expression scenario, and experiments were performed at least twice. Data shown in Table 3 are from a single representative experiment, and each strain listed is an independent ectopic expression mutant.

### Determination of mating type in *C. albicans.*

To determine the mating type of C. albicans strains, we PCR amplified the **a** and α alleles of each gene located within the *MTL* locus (*PAP, OBP, PIK, MTLa1, MTLa2, MTLα1, MTLα2*) [30]. PCR products for every **a** allele were seen in all strains tested; products for each α allele were seen in all strains except SS4 (unpublished data). PCR products for the *MTLa1* and *MTLα2* genes are shown in Figure 2.

### ChIP experiments.

Overnight cultures (200 ml) were grown in SCD+Urd for approximately 16 h at 25 °C to an OD_600_ of 0.4. Cells were formaldehyde cross-linked by adding formaldehyde (37%) to a 1% final concentration. Treated cultures were mixed by shaking and incubated for 15 min at room temperature. 2.5 M glycine was added to a final concentration of 125 mM, and treated cultures were mixed and incubated 5 min at room temperature. Cells were pelleted at 3,000 *g* for 5 min at 4 °C and washed twice with 100 ml of 4 °C TBS (20 mM TrisHCl [pH 7.6], 150 mM NaCl).

Spheroplasting and ChIP were carried out as previously described, with modifications [11,31]. Cell pellets were resuspended in 39 ml of Buffer Z (1 M sorbitol, 50 mM Tris-Cl [pH 7.4]), 28 μl of β-ME was added (14.3 M, final concentration 10 mM), and cells were vortexed. 20 μl of lyticase (Sigma, MO, United States) solution (2 mg/ml in Buffer Z) was added, and cell suspensions were incubated 15 min at 30 °C. Spheroplasted cells were then spun at 3,000 *g,* for 10 min, at 4 °C and resuspended in 500 μl of 4 °C lysis buffer (50 mM HEPES-KOH [pH 7.5],140 mM NaCl, 1 mM EDTA, 1% Triton X-100, 0.1% sodium deoxycholate) with protease inhibitors. All subsequent ChIP and wash steps were done at 4 °C. DNA was sheared by sonication ten times for 10 s at power setting 2 on a Branson 450 sonicator (http://www.bransonultrasonics.com/), incubating on ice for 2 min between sonication pulses. Extracts were clarified by centrifugation. A 50 μl aliquot of each extract was set aside as ChIP input material.

For the IP, 450 μl of lysis buffer was added to 50 μl of extract, and 5 μl of α-Wor1_Nterm_ antibody was added. α-Wor1_Nterm_ is an affinity-purified antibody generated against a peptide, QVLDKQLEPVSRRPHERER, located near the N terminus of Wor1 (Bethyl Laboratories, http://www.bethyl.com/). The IP was incubated for 2 h at 4 °C, with agitation. Then 50 μl of a 50% suspension of protein A-Sepharose Fast-Flow beads (Sigma, http://www.sigmaaldrich.com/) in lysis buffer was added to the IP and incubated 1.5 h at 4 °C with agitation. The beads were pelleted for 1 min at 3,000 *g*. After removal of the supernatant, the beads were washed with a series of buffers for 5 min for each wash: twice in lysis buffer, twice in high-salt lysis buffer (50 mM HEPES-KOH [pH 7.5], 500 mM NaCl, 1 mM EDTA, 1% Triton X-100, 0.1% sodium deoxycholate), twice in wash buffer (10 mM Tris-HCl [pH 8.0], 250 mM LiCl, 0.5% NP-40, 0.5% sodium deoxycholate, 1mM EDTA), and once in TE (10 mM Tris, 1 mM EDTA [pH 8.0]). After the last wash, 100 μl of elution buffer (50 mM Tris-HCl [pH 8.0], 10 mM EDTA, 1% SDS) was added to each sample, and the beads were incubated at 65 °C for 15 min. The beads were spun for 1 min at 10,000 *g,* and the supernatant was removed and retained. A second elution was carried out with 150 μl of elution buffer 2 (TE, 0.67% SDS) and eluates from the two elution steps were combined. For the ChIP input material set aside, SDS (1% final concentration) and 200 μl of TE were added. ChIP and input samples were incubated overnight at 65 °C to reverse the formaldehyde crosslinks. 250 μl of proteinase K solution (TE, 20 μg/ml glycogen, 400 μg/ml proteinase K) was added to each sample, and samples were incubated at 37 °C for 2 h. Samples were extracted once with 450 μl Tris buffer-saturated phenol/chloroform/isoamyl alcohol solution (25:24:1). 55 μl of 4 M LiCl and 1 ml of 100% ethanol (4 °C) were added and the DNA was precipitated for 1 h at 4 °C. The DNA was pelleted by centrifugation at 14,000 *g* for 15 min at 4 °C, washed once with cold 75% ethanol, and allowed to air dry. The samples were resuspended in 25 μl of TE containing 100 μg/ml RNaseA and incubated 1 h at 37 °C.

ChIPs were also carried out in experiments not shown using affinity-purified antibody generated against a peptide DDAVGNSSGSYYTGT, located at the C terminus of Wor1 (α-Wor1_Cterm_) (Bethyl Laboratories) [11]. ChIP was performed in WT opaque strains twice using α-Wor1_Nterm_ and three times using the α-Wor1_Cterm_ antibodies. Control ChIPs were performed in the *wor1*Δ/*wor1*Δ mutants using α-Wor1_Nterm_ once, and the α-Wor1_Cterm_ was used twice.

### DNA amplification and labeling.

ChIP-enriched DNA was amplified and fluorescence labeled as described [32]. Labeled DNA for each channel was combined and hybridized to arrays in Agilent hybridization chambers for 40 h at 65 °C, according to protocols supplied by Agilent (Agilent Technologies, http://www.agilent.com/). Arrays were then washed and scanned, using an Axon Instruments Genepix 4000A scanner.

### Tiling array design.

Approximately 185,000 60-mer oligo probes were designed across the entire *Candida* genome (contig20 haploid genome assembly) at approximately 80 bp intervals, excluding nonunique regions of the genome (see Protocol S1 for further information). Custom microarrays were manufactured by Agilent Technologies (Agilent Technologies). Array design and ChIP-chip data are available on GEO.

### Data analysis.

Arrays were blank subtraction normalized, interarray median normalized, and intra-array median normalized using Agilent ChIP Analytics 1.3 software (Agilent Technologies). After normalization, average ratios across replicate arrays (where relevant) were used for further analysis. After normalization, the single array error model was applied across replicate arrays (where relevant), to derive a *p*-value statistic to represent the probabilities that data at each spot occurred within experimental noise. A segment is a region of adjacent probes containing peaks of Wor1 enrichment, where the enrichment above input is considered to be statistically significant, based on the parameters set in the software. Using the ChIP Analytics software, the Whitehead Neighborhood Model was applied using default parameters as described [21] to map the segments according to their chromosomal positions. When comparing ChIP-chip experiments in WT opaque strains against *wor1*Δ/*wor1*Δ strains, or between α-Wor1 ChIP-chip experiments performed in WT opaque strains using the two different Wor1 antibodies, any overlapping segments were eliminated from further analysis.

Within each segment, we used ChIP Analytics software to identify the location of highest Wor1 enrichment (corresponding to the probe with the lowest P[ξ]-value). The positions of peaks were then assessed in relationship to ORFs throughout the C. albicans genome; an ORF was identified as being potentially regulated by Wor1 if there was a segment of Wor1 enrichment within the intergenic region immediately upstream of the given coding sequence.

## Supporting Information

Protocol S1 C. albicans Tiling Array Design (35 KB DOC)

Table S1 ORFs with Wor1 Bound at the Intergenic DNA Upstream of Their Coding Sequences ORFs potentially directly regulated by Wor1, as identified by ChIP-chip. ID: orf19# of each ORF with Wor1-enrichment in the intergenic region immediately upstream of its coding sequence. MinP[ξ]: the lowest P[ξ] value for a probe within each segment of Wor1 enrichment. As calculated by Chip Analytics, the P[ξ] statistic estimates the probability that a set of neighboring probes, within a window surrounding a given probe, are not enriched. Op/Wh expression ratios (log_2_): log_2_ ratios for each ORF, compiled from expression microarrays comparing white and opaque isogenic strains [3]. For each *MTL* mutant capable of white-opaque switching, opaque/white expression ratios were calculated for each ORF. These expression ratios were then averaged between all *MTL* genotypes examined. Values greater than 1 indicate the gene is up-regulated at least 2-fold in the opaque state; values less than −1 indicate the gene is up-regulated at least 2-fold in the white state. Gene name, S. cerevisiae orthologs, and description of each ORF were taken from the Candida Genome Database on 22 April 2007 (http://www.candidagenome.org/). Information was edited and supplemented by hand, based on current work, Pfam searches (http://www.sanger.ac.uk/Software/Pfam/), and GO annotations.
(97 KB XLS)

Table S2 Strains Used in This Study (40 KB XLS)

Table S3 Primers Used in This Study (26 KB XLS)

### Accession Numbers

Candida Genome Database (http://www.candidagenome.org/) accession numbers for the genes discussed in this article are *CZF1* (orf19.3127), *WOR2* (orf19.5992), *HAP3* (orf19.4647), orf19.4972, *CSR1* (orf19.3794), and *PHO23* (orf19.1759).

## Abbreviations

ChIP: chromatin immunoprecipitation
ChIP-chip: chromatin immunoprecipitation, analyzed genome-wide using microarrays
IP: immunoprecipitation
MTL: mating type-like
ORF: open reading frame
SCD+Urd: synthetic complete medium plus 2% glucose and 100 μg/ml uridine
WT: wild type
