# Supplementary material for: Interlocking Transcriptional Feedback Loops Control White-Opaque Switching in Candida albicans
Source: PLoS Biol. 2007 Sep 18;5(10):e256. doi: 10.1371/journal.pbio.0050256 (PMC1976629; doi:10.1371/journal.pbio.0050256)
Supplement: Supplementary file 1 [file pbio.0050256.sd001.doc]

**Protocol S1. *C. albicans* Tiling Array Design**

We designed custom ChIP-Chip arrays for *C. albicans* by tiling 182,900 probes of length 60bp across the ~14.3 Mb of sequence included in what, at the time of design, was the most recent build of the genome (Assembly 20 from Andre Nantel in April 2006). Rather than choose probes spaced uniformly, an effort was made to optimize four characteristics of the oligo set (uniqueness, GC content, self-annealing and sequence complexity), while still maintaining probe spacing that is close to uniform. We used a previously developed algorithm, ArrayOligoSelector[1], to score all possible probes for each of these four characteristics. These scores, as well as a penalty for a too-long or too-short distance to the neighboring probe, were integrated into a single score via a weighting scheme. Due to the lack of studies which systematically explore the importance of uniform spacing and each of the four probe characteristics on ChIP-chip quality, we chose weights based largely on intelligent guessing. Stronger weights were given to probe spacing, GC content and uniqueness than to self-annealing and sequence complexity. A Monte Carlo optimization was employed to search for the highest scoring probe set.

The highest scoring probe set found had median probe spacing (measured from oligo start to oligo start) of 80bp with a range of ~30bp to ~130bp:

The uniqueness of the probe set (blue curve) was significantly higher than that for the whole genome (red curve). A higher uniqueness score indicates a more unique probe that is less likely to be affected by cross-hybridization. Whereas only 27% of all possible probes are completely unique (uniqueness score of 0.0), 40% of the probes in our probe set are completely unique. Note that the tail of this distribution (uniqueness score < 35) has been truncated in the graph.

Finally, the GC content of the chosen oligos (blue curve) was much improved over the genome as a whole (red curve). While the average was kept similar to that of the whole genome (~35%), the variance was considerably reduced.

1. Bozdech Z, Zhu J, Joachimiak MP, Cohen FE, Pulliam B, et al. (2003) Expression profiling of the schizont and trophozoite stages of Plasmodium falciparum with a long-oligonucleotide microarray. Genome Biol 4: R9.
